# Head-to-Head Comparison Between [^18^F]FDG and [^68^Ga]Ga-Pentixafor PET/CT in Multiple Myeloma: A Systematic Review and Meta-Analysis

**DOI:** 10.3390/cancers18203305

**Published:** 2026-10-14

**Authors:** Domenico Albano, Slavko Tasevski, Marco Rapa, Cristina Nanni, Giorgio Treglia

**Affiliations:** 1 Nuclear Medicine, University of Brescia, 25121 Brescia, Italy; 2 Nuclear Medicine Department, ASST Spedali Civili di Brescia, 25123 Brescia, Italy; 3 Nuclear Medicine, University Institute for Positron Emission Tomography, 1000 Skopje, North Macedonia; slavko.tasevski@uipet.mk; 4 Department of Experimental, Diagnostic and Specialty Medicine, Alma Mater Studiorum, University of Bologna, 40138 Bologna, Italy; marco.rapa@studio.unibo.it; 5 Nuclear Medicine Unit, Istituto di Ricovero e Cure a Carattere Scientifico (IRCCS), Azienda Ospedaliero-Universitaria di Bologna, 40138 Bologna, Italy; cristina.nanni@aosp.bo.it; 6 Faculty of Biology and Medicine, University of Lausanne, 1015 Lausanne, Switzerland; giorgio.treglia@eoc.ch; 7 Division of Nuclear Medicine, Ente Ospedaliero Cantonale, 6500 Bellinzona, Switzerland; 8 Faculty of Biomedical Sciences, Università della Svizzera Italiana, 6900 Lugano, Switzerland

**Keywords:** PET/CT, FDG, multiple myeloma, pentixafor, CXCR4, PET, meta-analysis

## Abstract

**Background/objective**: Multiple myeloma (MM) is a hematological malignancy characterized by the proliferation of clonal plasma cells. While [^18^F]FDG PET/CT is widely established for assessing disease extent, bone involvement, and treatment response in MM, novel molecular imaging targets like CXCR4 (e.g., using [^68^Ga]Ga-Pentixafor) have emerged to offer potentially superior sensitivity and theranostic applications. The aim of this manuscript is to systematically review and compare the diagnostic performance, expressed as detection rates, of CXCR4-targeted PET/CT versus [^18^F]FDG PET/CT in patients with MM. **Methods**: A systematic literature search was conducted across major databases (PubMed/MEDLINE, Embase, and the Cochrane Library) for head-to-head comparative studies evaluating both [^18^F]FDG and [^68^Ga]Ga-Pentixafor PET/CT in MM patients. Risk of bias and study quality were assessed using the QUADAS-2 tool. **Results**: Eleven studies were collected, amounting to a total of 347 patients. A patient-based analysis showed that [^68^Ga]Ga-Pentixafor had a higher detection rate than [^18^F]FDG PET/CT (86.3%, 95% CI: 80.3–92.3% vs 60.9%, 95% CI: 42.5–79.4%). In a descriptive analysis, mean lesion SUVmax values reported across individual studies were generally higher with [^68^Ga]Ga-Pentixafor PET/CT than with [^18^F]FDG PET/CT. **Conclusions**: [^68^Ga]Ga-Pentixafor PET/CT may serve as a robust alternative or complementary imaging modality to [^18^F]FDG PET/CT in MM. It could also be helpful for selection in CXCR4-directed theranostic pathways, but more high-quality studies are warranted.

## Simple Summary

This systematic review and meta-analysis aimed to perform a head-to-head comparison of the diagnostic performance of [^18^F]FDG and [^68^Ga]Ga-Pentixafor PET/CT in multiple myeloma (MM). The detection rate of [^68^Ga]Ga-Pentixafor PET/CT was significantly higher than that of [^18^F]FDG PET/CT. Moreover, the mean radiotracer uptake value of MM lesions detected by [^68^Ga]Ga-Pentixafor PET/CT was significantly higher than that by [^18^F]FDG PET/CT. Future prospective, large-scale multicenter studies are warranted to further define its impact on long-term clinical staging, prognostic stratification, and therapy response monitoring.

## 1. Introduction

Multiple myeloma (MM) is a hematologic malignancy characterized by plasma cell proliferation in the bone marrow, often resulting in bone lesions, renal impairment, anemia, and hypercalcemia (“CRAB” criteria) [1]. Accurate assessment of disease burden, staging, and treatment response is critical for guiding therapeutic strategies and predicting patient outcomes [2]. Positron emission tomography/computed tomography (PET/CT) using 2-deoxy-2-[^18^F]fluoro-D-glucose ([^18^F]FDG) is currently the gold-standard imaging modality recommended by the International Myeloma Working Group (IMWG) [3]. It offers high specificity and provides vital functional data regarding metabolic activity [4,5,6]. However, [^18^F]FDG is not accurate in all patients and may present several diagnostic limitations. Due to low hexokinase-2 expression, up to 20% [7] in certain myeloma cell variants, physiological background activity in bone marrow, or indolent disease subtypes, [^18^F]FDG PET/CT can yield false-negative results in a notable subset of patients. Furthermore, inflammatory processes and recent corticosteroid or growth factor therapies can induce false-positive uptake or obscure malignant lesions, complicating response evaluation. To address these gaps, novel radiopharmaceuticals targeting specific molecular markers of plasma cells have emerged. Among these, target agents directed against C-X-C chemokine receptor type 4 (CXCR4), primarily labeled as [^68^Ga]Ga-Pentixafor, have shown significant diagnostic promise in several oncological [8] and hematological diseases [9,10]. CXCR4, a member of the G-protein-coupled chemokine receptor family, interacts with its ligands CXCL12 or stromal cell-derived factor (SDF-1), is highly expressed on myeloma cells, and plays a critical role in bone marrow homing and tumor growth while also offering theranostic potential thanks to its therapeutic twin molecules [^177^Lu]Lu- or [^90^Y]Y-Pentixather. As there is increasing interest among the scientific community in understanding whether [^68^Ga]Ga-Pentixafor could complement or substitute [^18^F]FDG PET/CT in MM, we performed the first systematic review and meta-analysis directly evaluating the diagnostic performance and clinical utility of CXCR4 PET/CT versus [^18^F]FDG PET/CT in MM.

## 2. Materials and Methods

### 2.1. Protocol

This systematic review and meta-analysis was conducted and reported in accordance with the Preferred Reporting Items for Systematic Reviews and Meta-Analyses (PRISMA) 2020 guidelines [11]. The completed PRISMA checklist is provided in Supplementary Table S1.

### 2.2. Search Strategy

Two independent nuclear medicine physicians (D.A., S.T.) systematically searched PubMed/MEDLINE, Embase, and the Cochrane Library from inception to 30 July 2026. Custom search strings were created for each database using combinations of terms related to (1) “PET” or “positron,” (2) “multiple myeloma,” (3) “CXCR” or “pentixafor,” and (4) “FDG” or “fluorodeoxyglucose.” To maximize search sensitivity, reference lists of relevant articles were manually screened. No restrictions were placed on language or publication date. Eligible studies required a head-to-head comparison of [^18^F]FDG PET/CT and CXCR4 PET/CT in at least 10 patients. We excluded review articles, preclinical or phantom studies, case reports, small case series (<10 patients), and studies with clear overlapping patient cohorts.

### 2.3. Data Extraction and Collection

Data extraction was independently executed by two investigators (D.A. and S.T.) using full texts and Supplementary Files. Extracted parameters included study characteristics (authors, year, country, design, funding, patient age/sex), imaging protocols (device type, administered activity, uptake window, scan interval, analysis method), and diagnostic performance expressed as detection rate (DR). Detection rate was selected as the primary endpoint rather than sensitivity and specificity due to the absence of a consistent reference standard across all included studies. Discrepancies were resolved through mutual consensus following data cross-checking.

### 2.4. Quality Assessment (Risk of Bias Assessment)

Risk of bias and applicability concerns were evaluated by two independent reviewers (S.T. and D.A.) using the Quality Assessment of Diagnostic Accuracy Studies—2 (QUADAS-2) tool [12]. Risk of bias was evaluated across four key domains (patient selection, index test, reference standard, and flow and timing), while applicability concerns were assessed for the first three domains. Each domain was judged as having a “low,” “high,” or “unclear” risk. Overall study quality was evaluated qualitatively across all domains, and no studies were excluded due to high risk of bias. Discrepancies between reviewers were resolved by consensus.

### 2.5. Statistical Analysis

The DRs of [^18^F]FDG and [^68^Ga]Ga-Pentixafor PET/CT were obtained from individual studies on a per-patient basis. A random-effects model was used for the statistical pooling of DRs. Pooled data were presented with 95% confidence intervals (95% CIs) and displayed using forest plots. Heterogeneity was estimated using the I-square index (I^2^); statistical heterogeneity among studies was present if I^2^ was higher than 50% [13]. Publication bias was assessed through the visual evaluation of a funnel plot and Egger’s test [14]. To account for the paired nature of the data (where both index tests were performed in the same cohort within each study), a direct comparative meta-analysis was conducted. Paired comparative metrics—specifically, the relative detection rate (RDR) comparing [^68^Ga]Ga-Pentixafor versus [^18^F]FDG—were synthesized using a random-effects meta-analysis model.

Statistical analysis was conducted using the open-source software OpenMeta Analyst^®^ (Brown University, Providence, RI, USA, version 10.12).

## 3. Results

### 3.1. Literature Search

**Figure 1:**
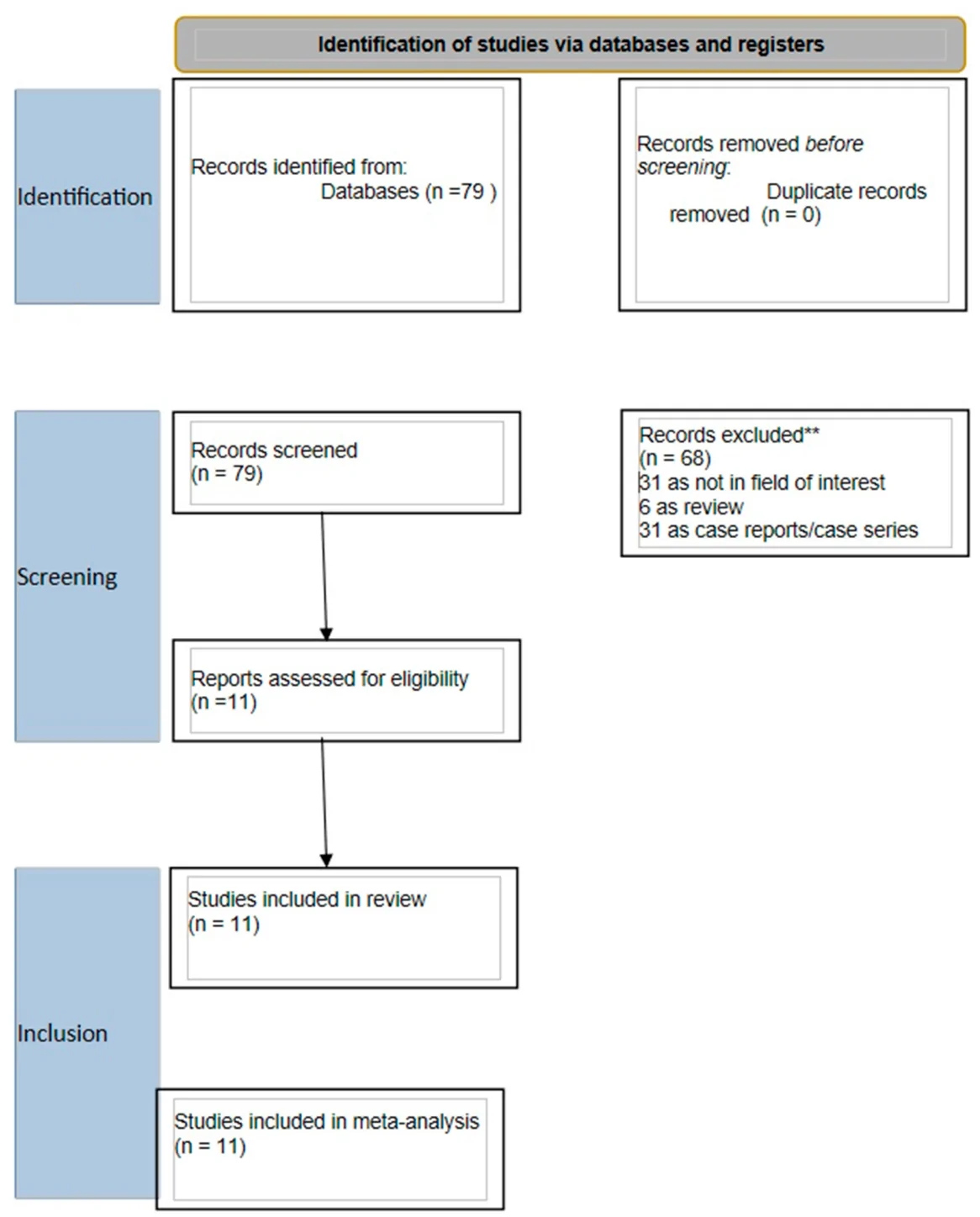
Flow-chart of studies included in our analysis.

Our initial literature search across the three selected databases yielded 79 records (Figure 1). We then screened these articles by title and abstract. This initial pass led to the exclusion of 68 articles for several reasons: 31 were outside our specific field of interest, 31 were either small case series or individual case reports, and six were identified as reviews. Ultimately, 11 studies [15,16,17,18,19,20,21,22,23,24,25] were deemed relevant for full-text evaluation and were subsequently included in this systematic review. Checking the references of these selected articles did not yield any additional records for inclusion.

### 3.2. Quality Assessment

**Figure 2:**
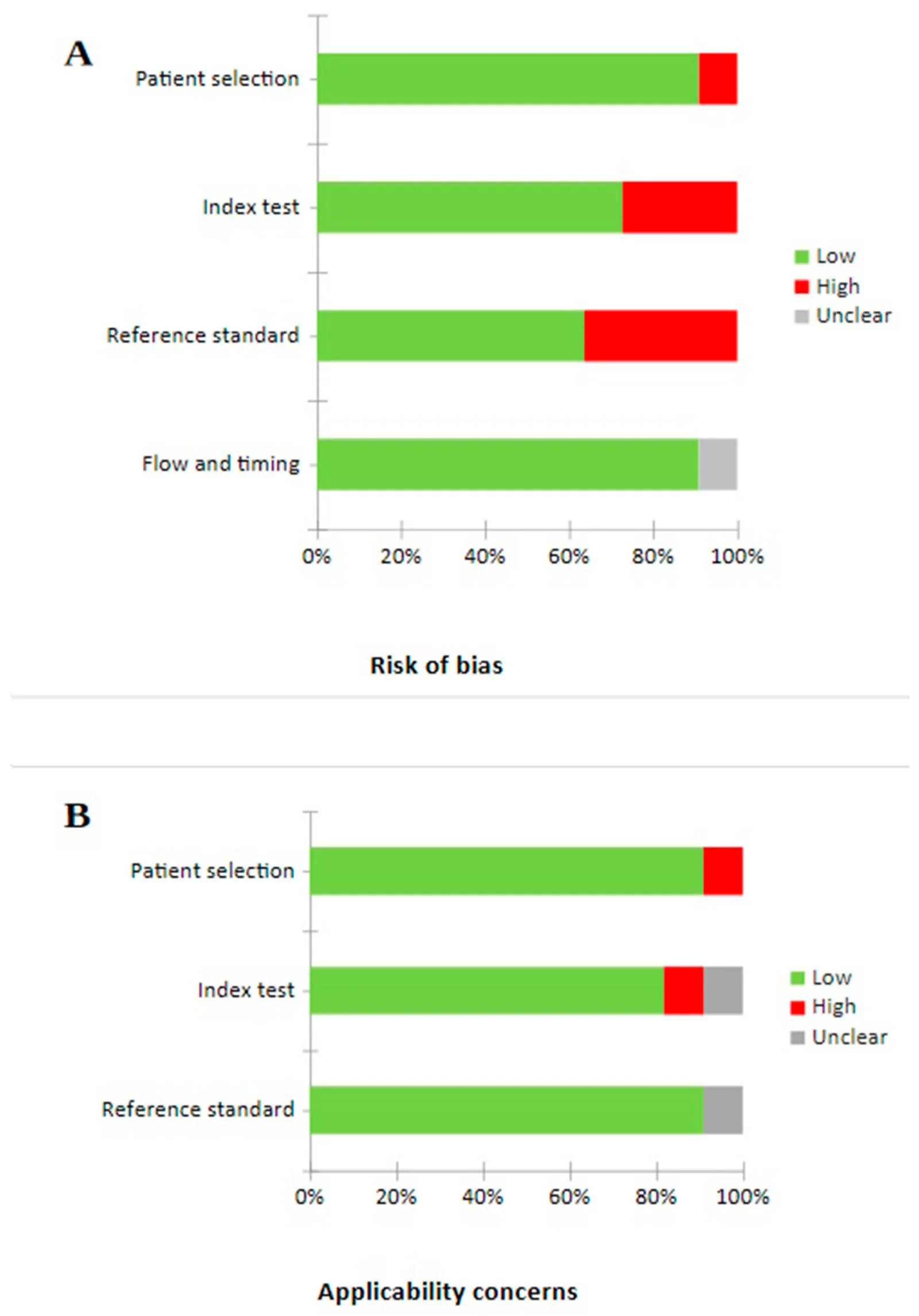
Representation of the QUADAS score of the included records considering risk of bias (**A**) and applicability concerns (**B**).

Risk of bias was assessed across seven items and is represented in Figure 2. No studies were excluded due to a high risk of bias. The most frequent potential sources of bias related to the “index test” and “reference standard” categories, primarily because several studies lacked clarity regarding blinded evaluations or failed to explicitly define the gold standard used for the final diagnosis.

### 3.3. Study and Patient Characteristics

The main characteristics of the 11 selected studies included in the systematic review are summarized in Table 1. Published between 2015 and 2026, the studies were primarily conducted in China (*n* = 4), Germany (*n* = 3), India (*n* = 2), Turkey (*n* = 1), and Austria (*n* = 1). Most adopted a retrospective design (*n* = 8), while three were prospective. Sample sizes ranged from 10 to 69 patients, yielding a total cohort across all studies, with a predominant male distribution overall. Patient median/average ages ranged from 56 to 72 years. The vast majority of studies (*n* = 9) received explicit funding support. Regarding imaging protocols, the reported time interval between [^68^Ga]Ga-Pentixafor and [^18^F]FDG PET assessments was generally short, typically within one to two weeks, though some studies allowed up to 26 or 30 days, and three studies did not report the specific interval. A critical point of methodological heterogeneity across all studies is the lack of a clear reference standard. None used histopathological confirmation for all lesions, which represents an intrinsic limitation. Instead, the final diagnosis was based on a consensus of standard imaging (CT, MRI, and FDG PET/CT itself), potentially introducing incorporation bias. This may overestimate the specificity of both tests and limit the strength of conclusions regarding true diagnostic accuracy.

### 3.4. Technical Aspects

Table 2 and Table 3 outline the technical parameters and image analysis criteria for both [^18^F]FDG and [^68^Ga]Ga-Pentixafor PET/CT acquisitions across the included studies. All studies utilized dedicated PET/CT scanners, with one study incorporating integrated PET/MRI. Three studies utilized analog systems, two of which employed them in combination with a digital system; this could potentially interfere with the DR, as digital systems exhibit higher sensitivity. For [^18^F]FDG PET/CT, administered weight-based doses ranged from 2.5 to 5.55 MBq/kg (or fixed activities of 176–380 MBq), with uptake times typically standardized to 60 min (range: 45–71.2 min). Positivity criteria for [^18^F]FDG were primarily defined as uptake greater than normal bone marrow, in only one as uptake greater than liver, or evaluated using standardized criteria such as the ImPETus system (Deauville Score > 3). For [^68^Ga]Ga-Pentixafor, tracer administration varied between 2 and 3 MBq/kg or fixed activities ranging from 90 to 205 MBq, with incubation times centered around 60 min (range: 35–60 min).

Positivity criteria for [^68^Ga]Ga-Pentixafor PET/CT commonly defined positive lesions as uptake exceeding normal bone marrow, liver, spleen, or contralateral background structures. All studies combined visual and semiquantitative image analyses, most frequently reporting SUVmax, followed by tumor-to-background ratios (TBRs). Instead, select studies incorporated volumetric metrics like MTV and TLG or related metrics (TBmG, TBmU). In descriptive assessments, individual studies consistently reported higher mean lesion SUVmax values for [^68^Ga]Ga-Pentixafor (ranging from 8.5 to 18.3) compared to [^18^F]FDG (ranging from 4.6 to 12.7).

### 3.5. Diagnostic Performances

**Figure 3:**
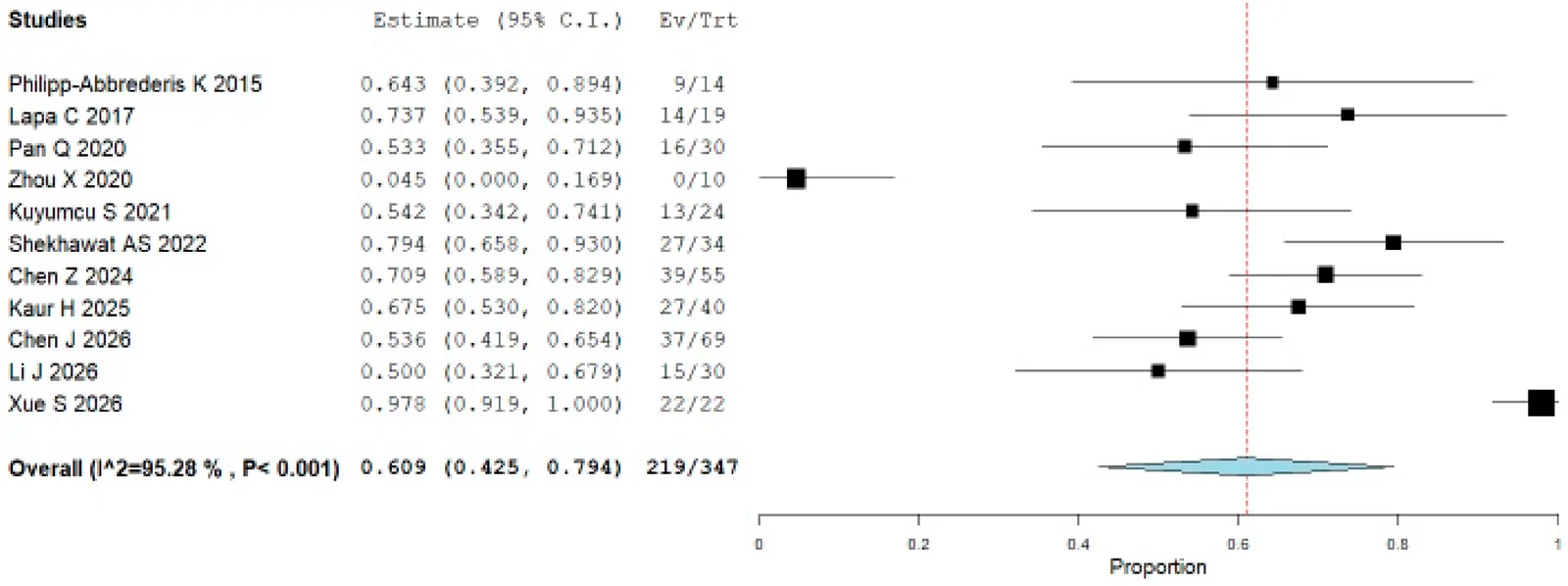
Forest plot of individual studies and pooled detection rate of [^18^F]FDG PET/CT [15,16,17,18,19,20,21,22,23,24,25].

**Figure 4:**
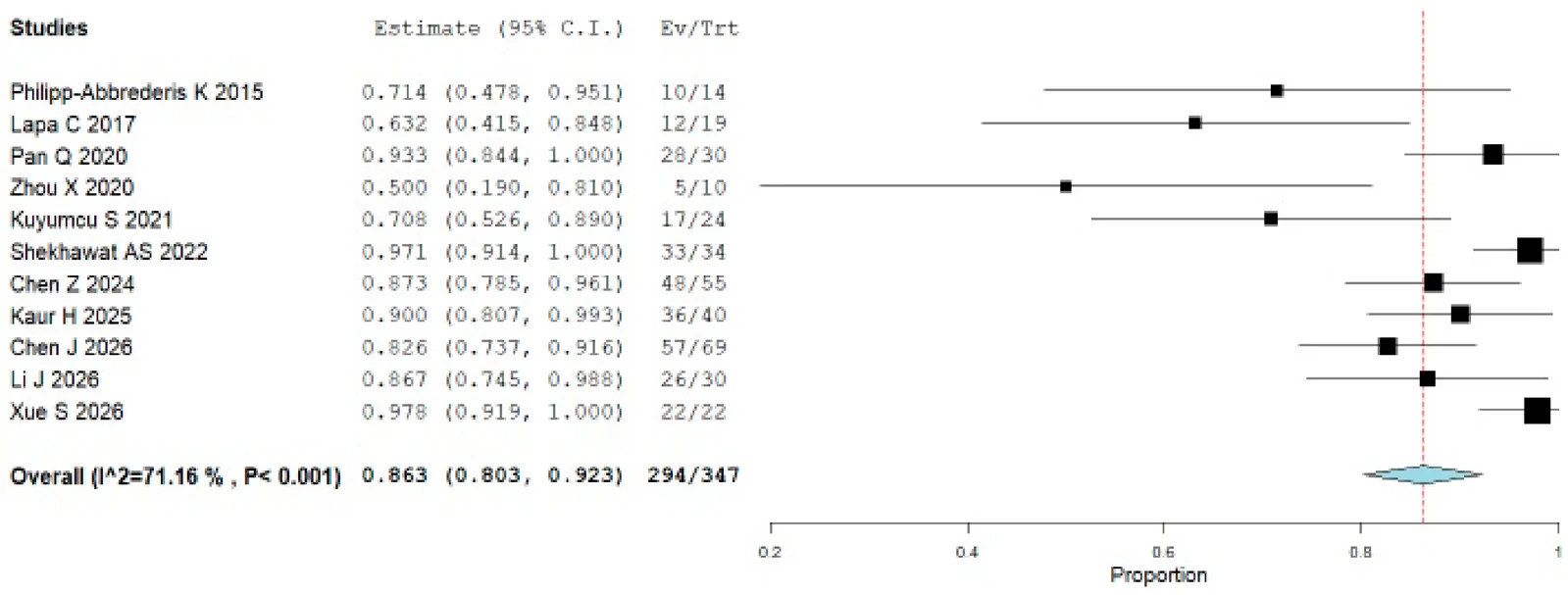
Forest plot of individual studies and pooled detection rate of [^68^Ga]Ga-Pentixafor PET/CT [15,16,17,18,19,20,21,22,23,24,25].

Across the 11 included studies, a total of 347 patients underwent paired head-to-head comparative evaluation with both [^18^F]FDG and [^68^Ga]Ga-Pentixafor PET/CT. Crude cumulative positive events were 219/347 (63.1%) for [^18^F]FDG and 294/347 (84.7%) for [^68^Ga]Ga-Pentixafor. Accounting for study weights and between-study variance using a random-effects meta-analysis model, the pooled overall detection rate was 60.9% (95% CI: 42.5–79.4%) for [^18^F]FDG PET/CT, exhibiting substantial heterogeneity (I^2^ = 95.28%, *p* < 0.001) (Figure 3), and 86.3% (95% CI: 80.3–92.3%) for [^68^Ga]Ga-Pentixafor PET/CT, showing moderate heterogeneity (I^2^ = 71.16%, *p* < 0–001) (Figure 4).

**Figure 5:**
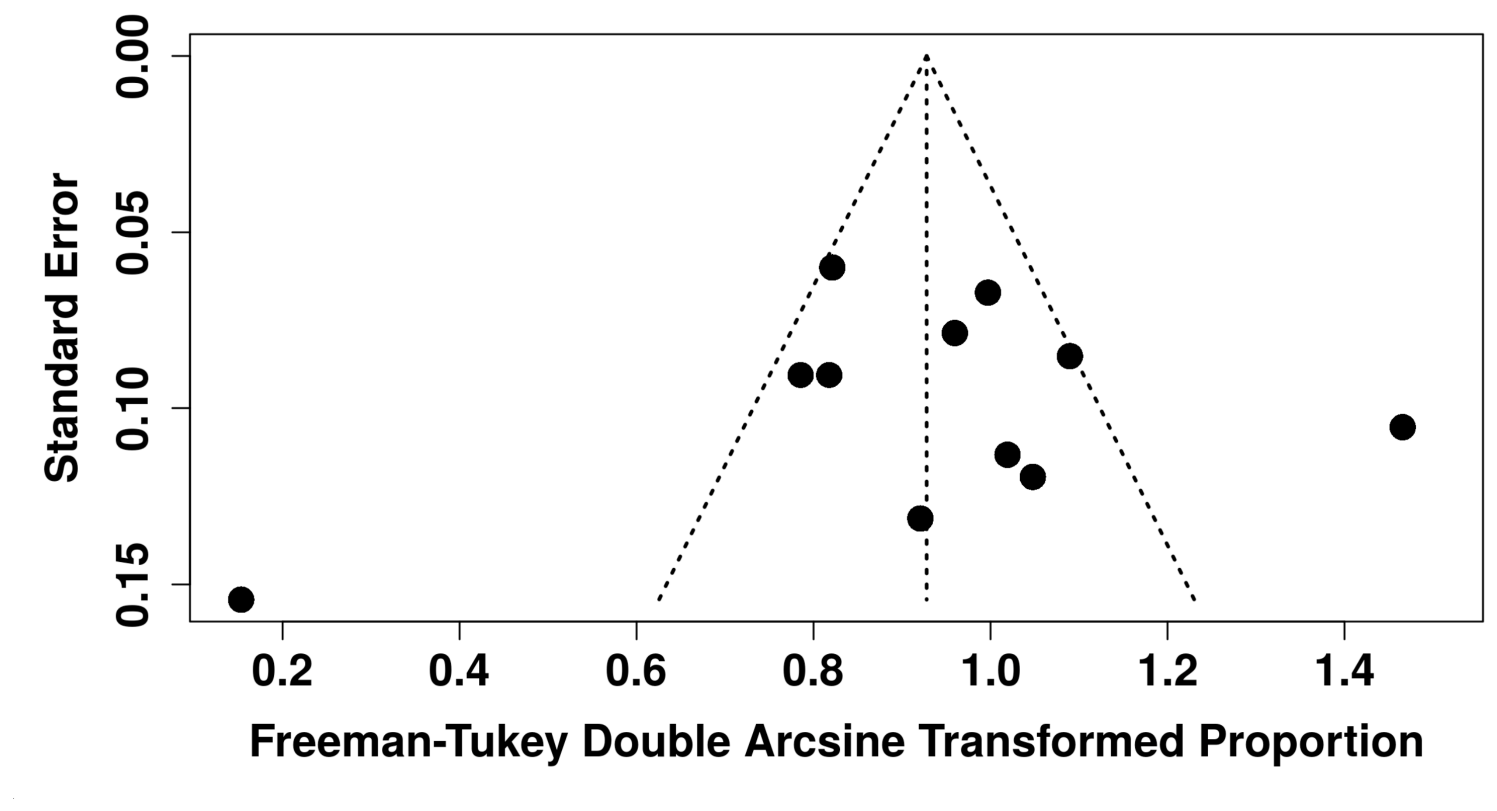
Funnel plot of publication bias for studies concerning [^18^F]FDG PET/CT.

The funnel plot for [^18^F]F-FDG does not indicate a potential publication bias (Figure 5). Egger’s test does not support the presence of funnel plot asymmetry (intercept: −0.71, 95% CI: −6.69–5.28, t: −0.232, *p*-value: 0.822). The raw, unweighted proportions of 219/347, or 63.1%, for [^18^F]F-FDG and 294/347 [84.7%] for [^68^Ga]Ga-Pentixafor represent crude cumulative counts across all included cohorts. In contrast, the reported meta-analytic estimates were derived using a random-effects model that accounts for study-level sample sizes, inverse-variance weighting, and between-study heterogeneity.

**Figure 6:**
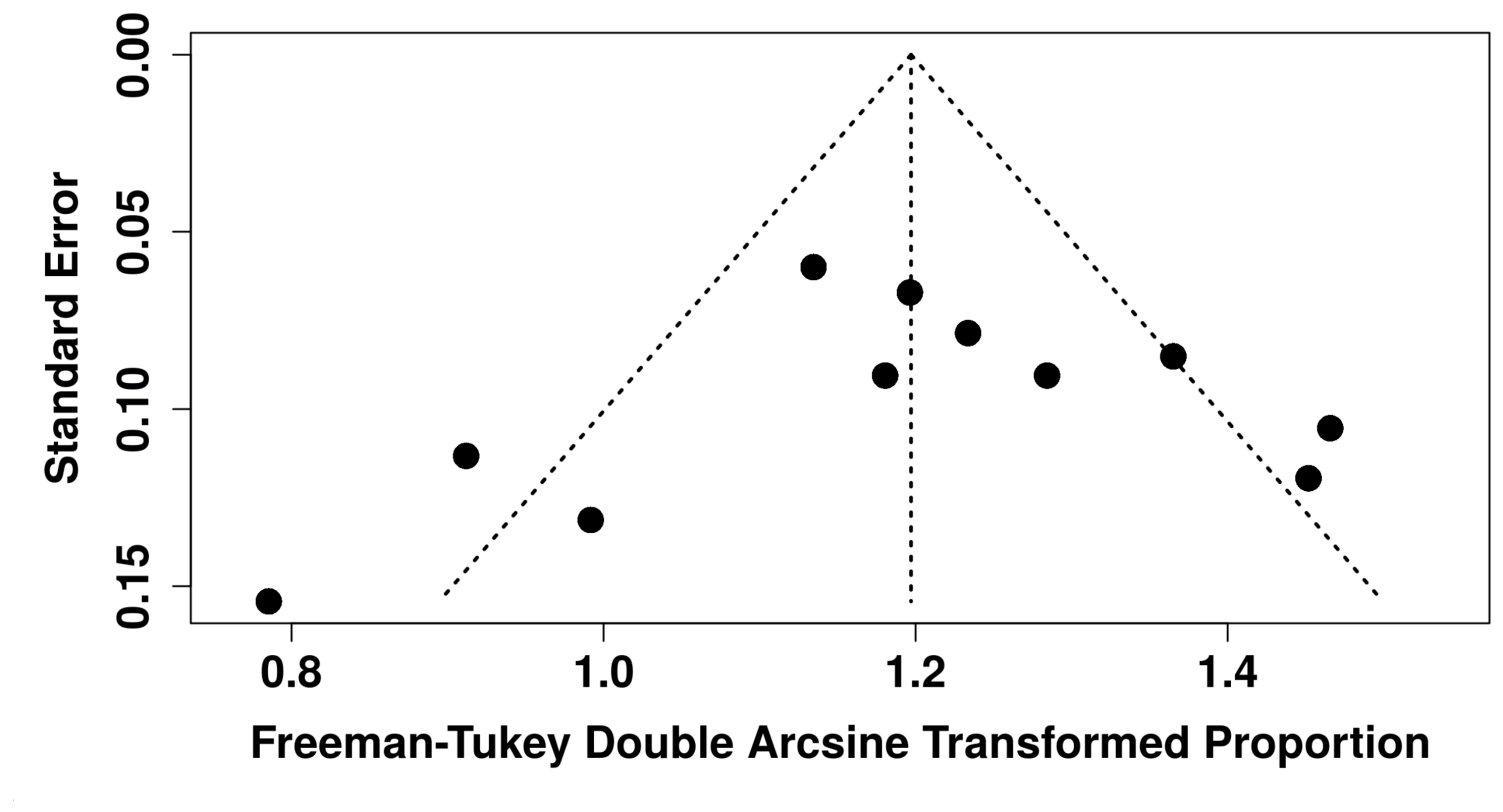
Funnel plot of publication bias for studies concerning [^68^Ga]Ga-Pentixafor PET/CT.

The funnel plot of [^68^Ga]Ga-Pentixafor does not point to a potential publication bias (Figure 6). Egger’s test excludes the presence of funnel plot asymmetry (intercept: −1.08, 95% CI: −5.31–3.15, t: −0.501, *p*-value: 0.628).

**Figure 7:**
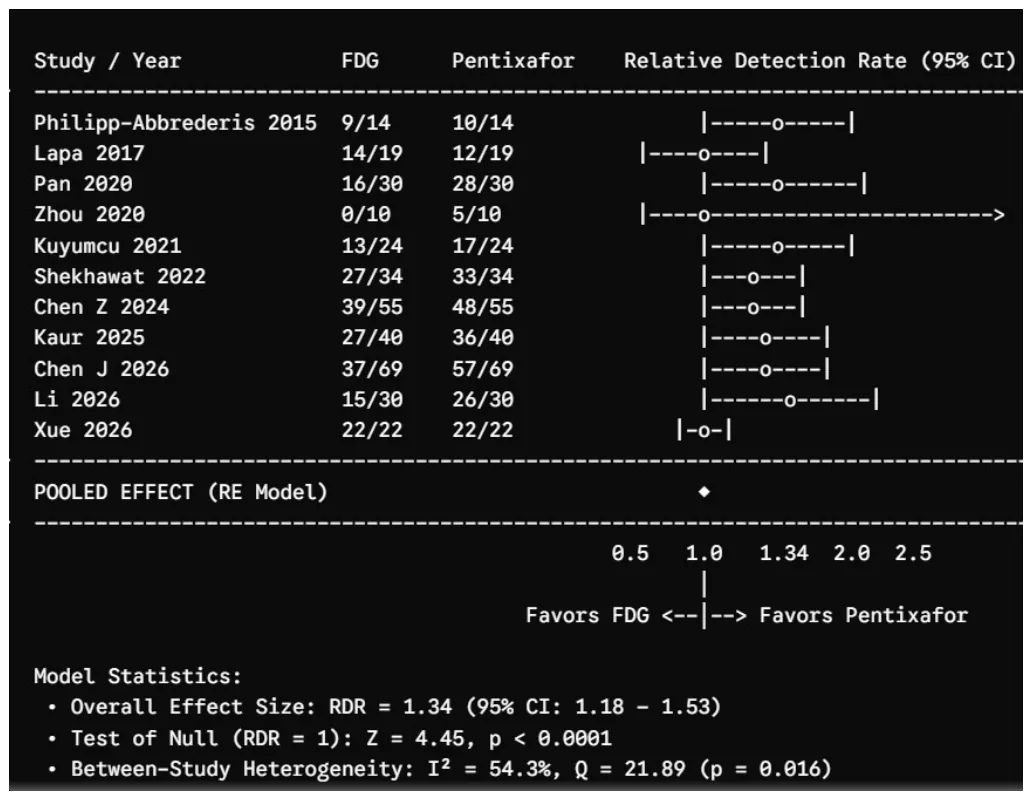
Funnel plot displaying study-specific relative detection rates (RDRs) with 95% confidence intervals (CIs) on a per-patient basis across 11 paired cohorts [15,16,17,18,19,20,21,22,23,24,25].

In the direct comparative paired meta-analysis (347 patients across 11 studies), [^68^Ga]Ga-Pentixafor demonstrated a statistically significant 34% higher detection rate compared with [^18^F]FDG in the same patient cohorts (RDR = 1.34, 95% CI: 1.18–1.53, *p* < 0.0001) (Figure 7). Between-study heterogeneity was moderate (I^2^ = 54.3%). This direct intra-patient comparison establishes a statistically significant superiority of [^68^Ga]Ga-Pentixafor over [^18^F]FDG for active lesion detection in multiple myeloma.

## 4. Discussion

MM is a complex, cytogenetically heterogeneous hematological malignancy in which precise staging, early detection of bone marrow involvement, and accurate monitoring of relapse are pivotal to clinical decision-making. While [^18^F]FDG PET/CT has long been established as a cornerstone imaging modality in the baseline assessment and response evaluation of MM, its diagnostic efficacy is constrained by metabolic variability and potential false-negative results. The emergence of alternative radiotracers seems to be mandatory to address these gaps [26]. This systematic review and meta-analysis provided a head-to-head comparison evaluating the diagnostic performance expressed as detection rates of [^18^F]FDG PET/CT versus CXCR4-directed PET/CT (specifically using [^68^Ga]Ga-Pentixafor) in patients with MM. This meta-analysis highlights a clear difference in overall detection rates between these two molecular imaging modalities across 347 evaluated patients. [^18^F]FDG PET/CT had a pooled detection rate proportion of 60.9% (95% CI: 0.425–0.794; 219/347 positive events), accompanied by substantial heterogeneity (I^2^ = 95.28%, *p* < 0.001). Instead, CXCR4 PET/CT achieved a significantly higher pooled detection rate proportion of 86.3% (95% CI: 0.803–0.923; 294/347 positive events), showing moderate statistical heterogeneity (I^2^ = 71.16%, *p* < 0.001). These findings indicate that CXCR4 PET/CT achieves a higher visual and quantitative disease DR for identifying active MM lesions compared to standard [^18^F]FDG PET/CT. While [^18^F]FDG PET remains the guideline-recommended standard imaging biomarker for assessing metabolic activity and treatment response, its sensitivity can be limited by low hexokinase-2 expression in certain MM clones, diffuse bone marrow involvement, or low-metabolic indolent disease. Conversely, C-X-C chemokine receptor type is highly expressed on the surface of plasma cells and plays a critical role in bone marrow homing and microenvironment crosstalk, providing a more reliable target for disease burden detection. Beyond visual analysis and detection rates, semiquantitative evaluation is more robust with [^68^Ga]Ga-Pentixafor than with [^18^F]F-FDG. Specifically, [^68^Ga]Ga-Pentixafor PET yields significantly higher mean SUV and SUV ratios (referenced to liver and blood pool) owing to superior lesion-to-background contrast.

Of course, comparison with SUVmax is not perfectly reproducible due to the presence of many potential sources of variability, including scanner type, acquisition and reconstruction protocols, and uptake time. However, the ratios can give an idea of radiotracer uptake.

Instead, other more sophisticated semiquantitative features, like volumetric features and/or radiomic texture features, have been only sparsely evaluated, lacking validated cutoff thresholds for risk stratification or differential diagnosis. The higher lesion diagnostic yield of CXCR4 PET/CT positions it as a highly promising complementary tool in MM management, particularly for staging diffuse involvement, clarifying ambiguous [^18^F]FDG findings, or evaluating minimal residual disease (MRD). Furthermore, endogenously high CXCR4 target expression and its high tumor-to-background ratio may hold critical therapeutic relevance, serving as a theranostic gateway to select candidates for CXCR4-targeted radioligand therapies (such as [^177^Lu]/[^90^Y]-Pentixather). There are, however, challenges with dynamic and variable CXCR4 expression levels, indicating that further investigation of receptor biology is required to fully understand the prognostic value and therapy response data. Small molecule alternatives to the peptidic agent, radiolabeled with either copper-64 or gallium-68, are undergoing preclinical evaluation and are likely to have a future clinical impact [27,28,29]. One point not clearly investigated is the potential role of PET/MRI in this scenario. Only in one study [15] were both PET/CT and PET/MRI scanners utilized without clear differences in terms of accuracy. However, the idea of combining metabolic features derived by PET and morphological/functional data from MRI could be a potential strength [30,31]. Even more evidence highlights the accurate impact of whole-body MRI in the study of MM [32], despite the combination of PET and MRI findings seeming to be complementary.

Another point that emerged in this research is the different criteria used in the interpretation of positive PET/CT scans, especially for FDG. Despite the presence of an international shared consensus [5], IMPetus criteria were not used universally for PET reporting: in five articles, BM was considered the standard of reference. Also, for [^68^Ga]Ga-Pentixafor, positivity criteria varied from uptake higher than bone marrow to uptake higher than surrounding background/contralateral tissue, liver/spleen background, or fixed cutoffs (SUVmax 2).

This heterogeneity in PET evaluation highlights the clinical need for standardized reporting criteria for CXCR4-targeted imaging, analogous to the IMPeTus criteria in FDG, to reduce inter-study variation in future trials.

Several methodological and clinical limitations must be acknowledged when interpreting findings of this head-to-head comparison: First, the overall pool of direct comparative literature remains relatively small, characterized by modest patient cohorts and potential selection bias; in particular, DRs were not analyzed in relation to the various staging systems (Durie-Salmon PLUS, R-ISS, R2-ISS) or according to the clinical–biological classification (Smouldering, relapse–refractory). In fact, in advanced stages, branching evolution leads to the coexistence of clones with different genomic and metabolic profiles, which heavily influence [^18^F]FDG DRs, causing significant differences even within the same patient. Other limitations include differences in radiotracer administration protocols, image acquisition timings, and variable SUV threshold definitions across participating centers, which introduce inter-study variability that may impact pooled diagnostic metrics; intra-individual and inter-individual variation in CXCR4 expression can occur over the course of treatment, meaning low expression in a subset of patients could yield false-negative results, necessitating continued reliance on [^18^F]FDG or hybrid imaging protocols; and the lack of a true reference standard. None of the included studies performed histopathological validation across all detected lesions. Diagnostics relied on composite clinical/imaging consensus, introducing potential incorporation bias. The detection rate metric is less robust than sensitivity or specificity, but without an accurate reference standard, their evaluation is not possible. All these reasons may explain the high heterogeneity, particularly for [^18^F]FDG.

## 5. Conclusions

In conclusion, [^68^Ga]Ga-Pentixafor PET/CT demonstrates a higher disease detection rate than [^18^F]FDG PET/CT in patients with MM. However, because true diagnostic accuracy metrics (such as sensitivity and specificity) cannot be formally established without a uniform, independent reference standard, and given the substantial heterogeneity across studies, these results should be interpreted cautiously as reflecting higher detection yield rather than proven overall diagnostic accuracy. These studies were evaluated at different stages of the natural history of a disease characterized by high intra- and inter-patient genotypic and metabolic heterogeneity. Future prospective, large-scale multicenter studies are warranted to further define its impact on long-term clinical staging, prognostic stratification, and therapy response monitoring.

## Figures and Tables

**Table 1:** Main features of the selected studies.

Interval Between [^68^Ga]Ga-Pentixafor and [^18^F] FDG	Average Age (Range)	M:F	N° pts	Study Design	Funding Source	Country	Year	First Author
nr	72 (51–84)	11:3	14	R	yes	Germany	2015	Philipp-Abbrederis K [15]
Within 2 weeks	62 (44–82)	23:12	35 *	R	yes	Germany	2017	Lapa C [16]
Within 1 week	59.1 (33–77)	19:11	30	P	yes	China	2020	Pan Q [17]
0–26 days	62	8:2	10	R	yes	Germany	2020	Zhou X [18]
Within 1 week	60.2	13:11	24	R	no	Turkey	2021	Kuyumcu S [19]
Within 1 week	57.5 (35–78)	21:13	34	R	yes	India	2022	Shekhawat AS [20]
nr	63 (48–80)	31:24	55	R	yes	China	2024	Chen Z [21]
nr	56 (34–81)	24:16	40	P	no	India	2025	Kur H [22]
1–12 days	64 (57–71.5)	40:29	69	P	yes	China	2026	Chen J [23]
Within 1 week	59 (33–88)	35:18	53 °	R	yes	China	2026	Li J [24]
Within 30 days	56 (42–71)	12:10	22	R	yes	Austria	2026	Xue S [25]

M: male; F: female; R: retrospective; P: prospective; nr: not reported; * 19 patients had both [^68^Ga]Ga-Pentixafor and [^18^F]FDG PET/CT; ° 30 executed both [^68^Ga]Ga-Pentixafor and [^18^F]FDG PET/CT.

**Table 2:** Technical characteristics of included studies concerning [^18^F]FDG.

[^18^F]FDG					Device [Analog or Digital]	First Author
SUVmax, Mean	Image Analysis	Mean Uptake Time (min)	Mean Injected Dose, MBq	Positivity Criterion		
5.5 (2.3–15.7)	Visual and semiquantitative analysis (SUVmax)	60	4.5/kg	uptake > normal BM	PET/CT Biograph mCT 64 (Siemens) [digital system] and PET/MRI Biograph mMR (Siemens)	Philipp-Abbrederis K [15]
11.6 (3.8–40.1) for intramedullary 9 (5–37.7) for extramedullary	Visual and semiquantitative analysis (SUVmax)	60	299 (256–340)	uptake > normal BM	PET/CT Biograph mCT 64 (Siemens) [digital system]	Lapa C [16]
7.1 (2.6–23.5)	Visual and semiquantitative analysis (TBmG)	71.2	5.5/kg	ImpeTus criteria (DS > 3)	PET/CT Biograph64 Truepoint TrueV (Siemens) [analog system] and Polestar m660, (SinoUnion) [digital system]	Pan Q [17]
na	Visual and semiquantitative analysis (SUVmax and TBR)	60	3–5/kg	ImpeTus criteria	PET/CT Biograph mCT 64 (Siemens) [digital system]	Zhou X [18]
12.7 (4.4–28.8)	Visual and semiquantitative analysis (TBmG)	60	2.5/kg	uptake > normal BM	PET/CT Biograph TruePoint TrueV (Siemens) [analog system]	Kuyumcu S [19]
4.6 (1.9–13.3)	Visual and semiquantitative analysis (SUVmax and TBR)	60	370	nr	PET/CT Discovery (GE) [digital system]	Shekhawat AS [20]
5.3 (3.4–7.3)	Visual and semiquantitative analysis (SUVmax and TBmU)	56.3	5.55/kg	uptake > normal BM	PET/CT Biograph mCT 64 (Siemens) [digital system]	Chen Z [21]
6.6	Visual and semiquantitative analysis (SUVmax and TBR)	45–60	176–380	ImPeTus criteria (DS > 3)	PET/CT Discovery 710 [analog system] and Discovery MIDR (GE) [digital system]	Kur H [22]
5.3	Visual and semiquantitative analysis (SUVmax, TBmV, TBmU, TLR)	60	240.5	Uptake > liver	PET/CT (Siemens) [digital system]	Chen J [23]
5.08 (2–16.09)	Visual and semiquantitative analysis (SUVmax and TBR)	55	3.7/kg	Uptake > normal BM	PET/CT Biograph Vision 600 (Siemens) [digital system]	Li J [24]
nr	Visual and semiquantitative analysis (SUVmax, MTV, TLG, TLC, Dmax)	60	298	ImPeTus criteria	PET/CT Biograph mCT (Siemens) [digital system]	Xue S [25]

nr: not reported; SUV: standardized uptake value; TBR: tumor-to-background ratio; BM: bone marrow; DS: Deauville score; TBmG: total bone marrow glycolysis; TBmU: total bone marrow uptake; TLR: tumor-to-liver ratio.

**Table 3:** Technical characteristics of included studies concerning [^68^Ga]Ga-Pentixafor.

[^68^Ga]Ga-Pentixafor					Device [Analog or Digital]	First Author
SUVmax, Mean	Image Analysis	Mean Uptake Time (h)	Mean Injected Dose, MBq	Positivity Criterion		
8.7 (1.4–33.7)	Visual and semiquantitative analysis (SUVmax)	60	90–205	uptake > normal BM	PET/CT Biograph mCT 64 (Siemens) [digital system] and PET/MRI Biograph mMR (Siemens)	Philipp-Abbrederis K [15]
16.4 (4.1–57.4) for intramedullary 14.9 (5–67.1) for extramedullary	Visual and semiquantitative analysis (SUVmax)	60	133 (43–165)	Uptake > surrounding normal tissue or contralateral structures	PET/CT Biograph mCT 64 (Siemens) [digital system]	Lapa C [16]
17 (6.1–74.5)	Visual and semiquantitative analysis (TBmU)	55.5	92.4 (40.7–170.2)	Uptake > liver or spleen	PET/CT Biograph64 Truepoint TrueV (Siemens) [analog system] and Polestar m660, (SinoUnion) [digital system]	Pan Q [17]
na	Visual and semiquantitative analysis (SUVmax and TBR)	60	2–3/kg	uptake > normal BM	PET/CT Biograph mCT 64 (Siemens) [digital system]	Zhou X [18]
18.3 (3.5–55.9)	Visual and semiquantitative analysis (TBmU)	60	185	Uptake > surrounding normal tissue or contralateral structures	PET/CT Biograph TruePoint TrueV (Siemens) [analog system]	Kuyumcu S [19]
8.5 (1.8–35.3)	Visual and semiquantitative analysis (SUVmax and TBR)	60	148–185	nr	PET/CT Discovery (GE) [digital system]	Shekhawat AS [20]
12 (5.1–16.1)	Visual and semiquantitative analysis (SUVmax and TBmU)	55	112.6	uptake > normal BM	PET/CT Biograph mCT 64 (Siemens) [digital system]	Chen Z [21]
17.3	Visual and semiquantitative analysis (SUVmax and TBR)	35–45	110–150	Uptake ≥ twice liver for diffuse uptake Uptake > controlateral structure for focal uptake	PET/CT Discovery 710 [analog system] and Discovery MIDR (GE) [digital system]	Kur H [22]
13.5	Visual and semiquantitative analysis (SUVmax, TBmV, TBmU, TLR)	60	122.1	Uptake > liver	PET/CT (Siemens) [digital system]	Chen J [23]
14.84 (2.72–128.27)	Visual and semiquantitative analysis (SUVmax and TBR)	60	148	Uptake > normal BM	PET/CT Biograph Vision 600 (Siemens) [digital system]	Li J [24]
nr	Visual and semiquantitative analysis (SUVmax, MTV, TLG, TLC, Dmax)	60	132		PET/CT Biograph mCT (Siemens) [digital system]	Xue S [25]

nr: not reported; SUV: standardized uptake value; TBR: tumor-to-background ratio; BM: bone marrow; TBmU: total bone marrow uptake; TLR: tumor-to-liver ratio.

## Data Availability

The datasets used and analyzed during the current study are available from the corresponding author on reasonable request.

## Supplementary Materials

The following supporting information can be downloaded at https://www.mdpi.com/article/10.3390/cancers18203305/s1, Table S1: PRISMA 2020 checklist. Reference [33] is cited in the Supplementary Materials.
